# Supplementary material for: Content-rich biological network constructed by mining PubMed abstracts
Source: BMC Bioinformatics. 2004 Oct 8;5:147. doi: 10.1186/1471-2105-5-147 (PMC528731; doi:10.1186/1471-2105-5-147)
Supplement: Additional File 5 — The original Chilibot query results of the term "long-term potentiation (LTP)" and 22 other terms, limiting the latest references analyzed to the years 1990, 1995, 2000, and 2004. [file 1471-2105-5-147-S5.bz2 › chilibotAdditionalFile5/ltp2000/html/LTP_TRKB.html]

 


 **LTP** and **TRKB** 
  
Found 18 abstracts in PubMed,  **18 abstracts were retrieved and analyzed**.  


---

 Search Google  |
 PDF files only 
|  EDU domain only 

---

**Interactive relationship** (e.g. stimulation, inhibition, etc)

- Inhibition of p75NTR does not detectably reduce  **LTP**  or affect presynaptic function, but analyses of newly generated  **trkB**  mutants implicate  **TrkB** .  Ref: 10995833 J Neurosci, 2000
- **TrkB**  IgG or K252a itself completely blocked  **LTP** , suggesting that endogenous BDNF or another  **TrkB**  ligand plays a role in  **LTP**  in the developing visual cortex.  Ref: 9254683 J Neurosci, 1997
- Moreover, normal  **LTP**  is generated in the mutant with reduced  **TrkB**  by a depolarization low frequency stimulation pairing protocol that puts minimal demands on presynaptic terminal function.  Ref: 10995833 J Neurosci, 2000
- Moreover, the induction of  **trkB**  and trkC mRNAs was attenuated by sodium pentobarbital, which interferes with the durability of  **LTP** .  Ref: 9191102 Brain Res Mol Brain Res, 1997
- A  **TrkB**  IgG fusion protein, which scavenges endogenous BDNF, reduced the synaptic responses to tetanus as well as the magnitude of  **LTP**  in adult hippocampus.  Ref: 8649517 Nature, 1996
- The  **TrkB**  Ab treated slices showed significant deficits in  **LTP**  induced by either theta burst stimulation TBS or pairing.  Ref: 9331355 Neuron, 1997
- The application of a  **TrkB**  IgG 30 min after  **LTP**  induction caused previously potentiated synaptic transmission to return to baseline levels, indicating that  **TrkB**  ligands are required to maintain  **LTP**  for up to 1 hr after induction.  Ref: 9331355 Neuron, 1997
- Activation of  **trkB**  also modulates hippocampal long term potentiation  [ **LTP** ] , in which postsynaptic N methyl D aspartate glutamate receptors play a key role.  Ref: 9223337 Proc Natl Acad Sci U S A, 1997
- **TrkB**  reduction affects presynaptic function and reduces the ability of tetanic stimulation to induce  **LTP** .  Ref: 10995833 J Neurosci, 2000
- Thus, both BDNF and its receptor  **trkB**  are induced in granule cells by stimulation that produces durable  **LTP** , suggesting that this neurotrophin and its receptor play an important role in memory formation and may be suitable targets for the development of cognitive enhancing drugs in the treatment of diseases, such as Alzheimer s.  Ref: 9191102 Brain Res Mol Brain Res, 1997
- Incubation of hippocampal slices with  **TrkB**  Ab had no effect on basal synaptic transmission, short term plasticity, or  **LTP**  induced by several trains of tetanic stimulation.  Ref: 9331355 Neuron, 1997
- Induction of long term potentiation  [ **LTP** ]   **LTP**  in the dentate gyrus of awake rats triggered a rapid 2 hour elevation in tyrosine kinase receptor  **trkB**  and trkC gene expression and a delayed 6 24 hour increase in brain derived neurotrophic factor BDNF and neurotrophin 3 NT 3 gene expression.  Ref: 8725345 J Comp Neurol, 1996
- The present results demonstrate that Trk B  [ **TRKB** ]  activation is required for the long term maintenance, > 30 min, of both  **LTP**  and  **LTP**  after LTD at P17.  Ref: 10762369 Eur J Neurosci, 2000
- At P23, a higher concentration of  **TrkB**  IgG was necessary to impair  **LTP** .  Ref: 10762369 Eur J Neurosci, 2000
- Consistent with this, elimination of  **TrkB**  in postsynaptic neurons does not affect  **LTP** .  Ref: 10995833 J Neurosci, 2000
- Shc binding site in the  **TrkB**  receptor is not required for hippocampal long term potentiation  [ **LTP** ] .  Ref: 10699438 Neuropharmacology, 2000
- The late phase of  **LTP**  2 3 hr was also significantly impaired in slices pretreated with  **TrkB**  Ab or a  **TrkB**  IgG.  Ref: 9331355 Neuron, 1997
- However, both  **TrkB**  IgG fusion proteins and BDNF antibody similarly reduced  **LTP** , suggesting that only BDNF but no other ligands of the  **TrkB**  receptor are likely to be involved in  **LTP**  induction.  Ref: 10479698 J Neurosci, 1999
- Taken together, these results indicate that endogenous Trk B  [ **TRKB** ]  ligands are necessary for the expression of  **LTP**  but not LTD at a critical time during postnatal cortical development.  Ref: 10762369 Eur J Neurosci, 2000
- This work has identified particular ITFs and neurotrophin systems tyrosine receptor kinase B, Trk B  [ **TRKB** ]  system that may be involved in stabilizing long lasting  **LTP** .  Ref: 10080384 Prog Neurobiol, 1999
- **TrkB**  expression in dentate granule cells is associated with a late phase of long term potentiation  [ **LTP** ] .  Ref: 9191102 Brain Res Mol Brain Res, 1997

**Parallel relationship** (e.g. studied together, co-existance, homology, etc.)

- Slices from BDNF knock out animals or slices treated with  **TrkB**  antibodies failed to exhibit L  **LTP** , indicating that  **TrkB**  ligands participate in extending synaptic enhancement from a short lasting to a long lasting form.  Ref: 9704996 NeuropharmacologyNeuropharmacology, 1997
- To test whether the Shc site activated signaling pathway, which has been shown to be important for neuronal survival in vivo, also plays a role in processes like long term potentiation  [ **LTP** ]   **LTP** , we have generated a mouse strain carrying a mutation in the Shc binding site of the  **TrkB**  receptor.  Ref: 10699438 Neuropharmacology, 2000
- Slices exposed to the same number of inducing stimuli, delivered either as TBS or as a single 100 Hz epoch, only exhibited  **TrkB**  sensitive  **LTP**  when TBS was used, indicating that the temporal pattern of stimulation determines the neurotrophin dependence.  Ref: 9331355 Neuron, 1997
- We examined the role of  **TrkB**  ligands in hippocampal long term potentiation  [ **LTP** ]   **LTP**  using function blocking  **TrkB**  antiserum Ab and Trk IgG fusion proteins.  Ref: 9331355 Neuron, 1997
- In developing hippocampus, the expression of brain derived neurotrophic factor BDNF and its receptor  **TrkB**  increases in parallel with the ability to undergo long term potentiation  [ **LTP** ]   **LTP** .  Ref: 8649517 Nature, 1996
- Trk B  [ **TRKB** ]  signalling controls  **LTP**  but not LTD expression in the developing rat visual cortex.  Ref: 10762369 Eur J Neurosci, 2000
- We found that homo and heterozygous mutant mice show no difference in the induction rate or magnitude of E  **LTP**  and L  **LTP**  induced by theta burst or tetanus stimulation, suggesting that the Shc binding site in the  **TrkB**  receptor and its downstream activated signaling cascade is not involved in hippocampal synaptic plasticity.  Ref: 10699438 Neuropharmacology, 2000
- We have examined whether BDNF or other  **TrkB**  ligands participate in L  **LTP**  in two ways by examining transgenic mice which lack BDNF or by acutely blocking  **TrkB**  function using function blocking antibodies.  Ref: 9704996 NeuropharmacologyNeuropharmacology, 2000
- Receptors for these neurotrophins have been cloned and characterized and we investigated whether  **LTP**  alters the expression of two neurotrophin receptors,  **trkB**  BDNF receptor and trkC NT 3 receptor in dentate granule neurons of the hippocampus using in situ hybridization analysis.  Ref: 9191102 Brain Res Mol Brain Res, 1997
- Here, we examined the contribution of endogenous Trk B  [ **TRKB** ]  activation to the regulation of different forms of synaptic plasticity including long term potentiation  [ **LTP** ]   **LTP** , long term depression LTD and  **LTP**  after LTD in the developing visual cortex.  Ref: 10762369 Eur J Neurosci, 2000
- The role of brain derived neurotrophic factor receptors in the mature hippocampus modulation of long term potentiation  [ **LTP** ]  through a presynaptic mechanism involving  **TrkB** .  Ref: 10995833 J Neurosci, 2000
- Thus, BDNF appears to act through  **TrkB**  presynaptically... to modulate  **LTP** .  Ref: 10995833 J Neurosci, 2000
- Brain derived neurotrophic factor BDNF and its receptor  **TrkB**  regulate both short term synaptic functions and long term potentiation  [ **LTP** ]   **LTP**  of brain synapses, raising the possibility that BDNF  **TrkB**  may be involved in cognitive functions.  Ref: 10571233 Neuron, 1999
- Because the reagents used in acute experiments react not only with BDNF but also with neurotrophin 4 5 NT4 5 and neurotrophin 3 NT3, we examined the involvement of these neurotrophins in  **LTP**  using two highly specific, function blocking monoclonal antibodies against BDNF and NT3, as well as a  **TrkB**  IgG fusion protein.  Ref: 10479698 J Neurosci, 1999
- Neurotrophins and time different roles for  **TrkB**  signaling in hippocampal long term potentiation  [ **LTP** ] .  Ref: 9331355 Neuron, 1997
